# Diabetes care cascade in Ukraine: an analysis of breakpoints and opportunities for improved diabetes outcomes

**DOI:** 10.1186/s12913-020-05261-y

**Published:** 2020-05-11

**Authors:** Robyn Margaret Stuart, Olga Khan, Romesh Abeysuriya, Tetyana Kryvchun, Viktor Lysak, Alla Bredikhina, Nina Durdykulyieva, Volodymyr Mykhailets, Elvira Kaidashova, Olena Doroshenko, Zara Shubber, David Wilson, Feng Zhao, Nicole Fraser-Hurt

**Affiliations:** 1 Department of Mathematical Sciences, University of Copenhagen, Universitetsparken 5, København Ø, 2300 Copenhagen, Denmark; 2 The World Bank Group, Washington, DC USA; 3 Monash University, Melbourne, Australia; 4 Department of Health, Poltava, Ukraine

**Keywords:** Diabetes, Care cascades, Optimization, Modeling, Service delivery

## Abstract

**Background:**

Diabetes is one of the leading causes of poor health and high care costs in Ukraine. To prevent diabetes complications and alleviate the financial burden of diabetes care on patients, the Ukrainian government reimburses diabetes medication and provides glucose monitoring, but there are significant gaps in the care continuum. We estimate the costs of providing diabetes care and the most cost-effective ways to address these gaps in the Poltava region of Ukraine.

**Methods:**

We gathered data on the unit costs of diabetes interventions in Poltava and estimated expenditure on diabetes care. We estimated the optimal combination of facility-based and outreach screening and investigated how additional funding could best be allocated to improve glucose control outcomes.

**Results:**

Of the ~ 40,000 adults in diabetes care, only ~ 25% achieved sustained glucose control. Monitoring costs were higher for those who did not: by 10% for patients receiving non-pharmacological treatment, by 61% for insulin patients, and twice as high for patients prescribed oral treatment. Initiatives to improve treatment adherence (e.g. medication copayment schemes, enhanced adherence counseling) would address barriers along the care continuum and we estimate such expenditures may be recouped by reductions in patient monitoring costs. Improvements in case detection are also needed, with only around two-thirds of estimated cases having been diagnosed. Outreach screening campaigns could play a significant role: depending on how well-targeted and scalable such campaigns are, we estimate that 10–46% of all screening could be conducted via outreach, at a cost per positive patient identified of US$7.12–9.63.

**Conclusions:**

Investments to improve case detection and treatment adherence are the most efficient interventions for improved diabetes control in Poltava. Quantitative tools provide essential decision support for targeting investment to close the gaps in care.

## Background

The global burden of diabetes is enormous, with an estimated 1.6 million deaths directly caused by diabetes in 2016, and another 2.2 million deaths attributable to high blood glucose [1]. The World Health Organization (WHO) estimates that diabetes was the seventh leading cause of death in 2016, and projections indicate that the burden is likely to continue growing. For Ukraine, as for many other lower-middle income countries (LMICs), this is placing considerable strain on already fragile health systems [2]. Diabetes is expensive to monitor and treat, and often occurs alongside multiple co-morbidities that require long-term health management. Globally, the costs associated with diabetes have been estimated at US$827 billion annually [3, 4], or more than US$1.7 trillion over 2011–2030 including indirect costs [5].

Minimizing costs of diabetes complications necessitates preventing complication via early diagnosis and consistent care and treatment. However, undiagnosed and untreated diabetes remain significant problems globally [6]. Along the entire diabetes care pathway from initial screening to sustained glucose control, there are many points at which patients can be lost, or where clinical outcomes may turn out poorer than desired. Late diagnosis, as well as poor glucose control while in diabetes care cause serious health risks, disabilities and costs in the health and social sectors. It is therefore imperative to ask whether there may be scope for improvements in the delivery of services designed to guide patients through the continuum of care. To address this question, it can be helpful to measure what proportion of the population has progressed to each consecutive stage in the care continuum; this kind of cascade analysis has been effectively applied to many areas of health, including to diabetes care in the U.S. [7] and in South Africa [8].

In this study, we build on previous work [9] that estimated the state of the type 2 diabetes cascade for Poltava, an oblast (region) of Ukraine with approximately 1.4 m inhabitants, which forms part of a World Bank support project to strengthen NCD programs in the Ukraine. This recent study identified two key breakpoints in the cascade: case detection, with about a third of estimated type 2 diabetes cases missing from the diabetes register, and glucose control among HbA1C monitored patients, with only around one in four patients achieving the glycated hemoglobin target level of ≤7.0%. Several Ukraine studies have identified the cost of diabetes medication and monitoring as important barriers to diabetes care [10–12]. Our main goals in this study are to investigate options for improving these two key breakpoints – diagnosis and glucose control – by using a mathematical model for optimal resource allocation.

The protocols and practices in place for the provision of diabetes care along the type 2 diabetes cascade in Poltava region are governed by the national guidelines issued by the Ministry of Health in 2012 [13]. The annual Health Index Surveys suggest that a portion of the population does not use the care provision at health facilities, indicating that more outreach services may be required to find people in need of care [14]. For instance, only 34% of Poltava adults sought out-patient care in case of sickness in 2017. Taking these protocols and survey findings as our starting point, we: (1) gather data on the unit costs of each of the interventions that form part of these protocols and practices and use these to estimate the cost of the response to type 2 diabetes in Poltava region in 2017; (2) focus on case detection, and investigate the optimal combination of facility-based and outreach screening; and (3) focus on adherence support interventions, and investigate how additional funding could best be allocated to get the maximum possible amount of people with sustained glucose control.

## Methods

### Patient pathways through type 2 diabetes care in Poltava region

**Fig. 1:**
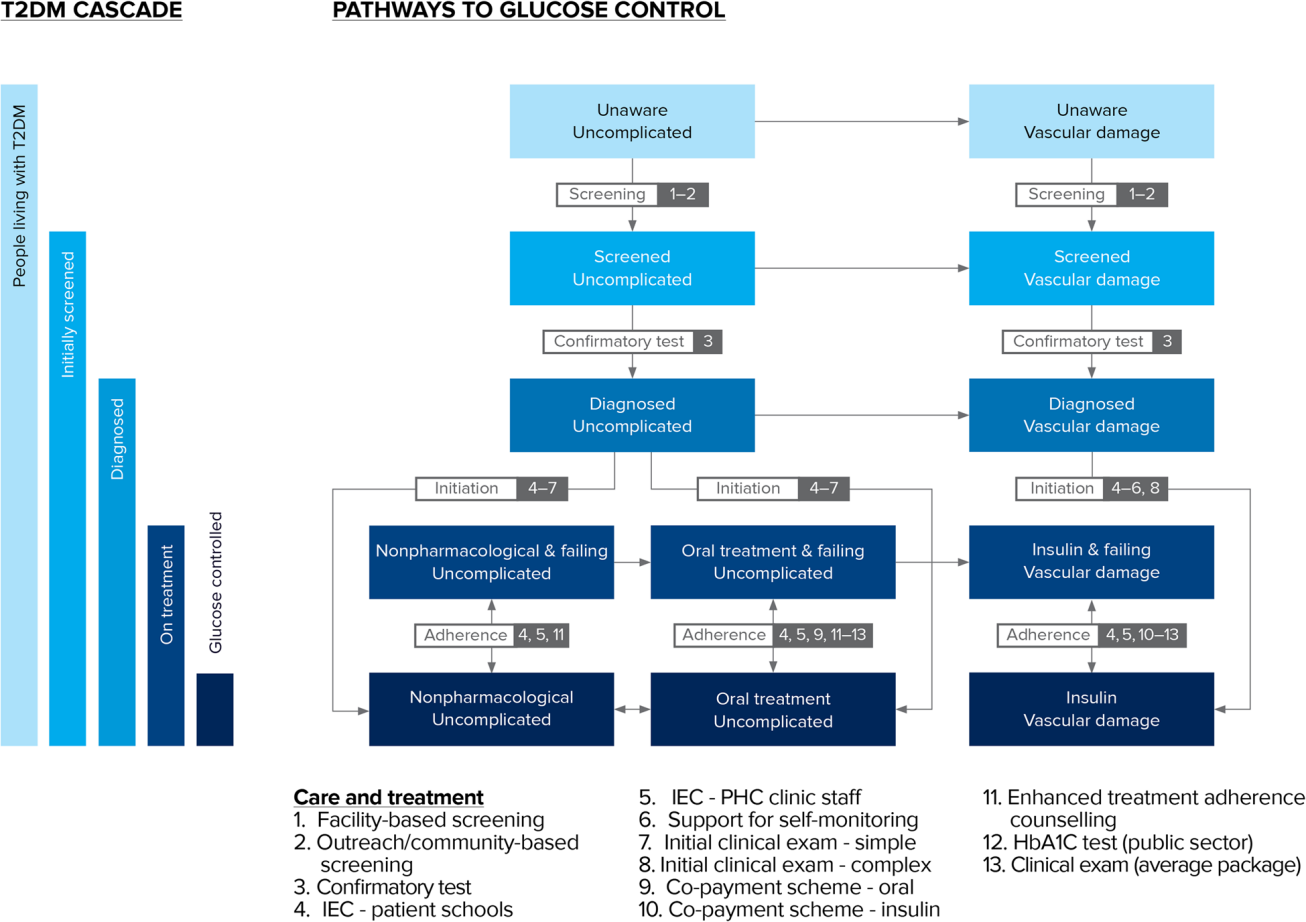
Schematic indicating the pathways through care for type 2 diabetes cases in Poltava region, Ukraine

Figure 1 summarizes the pathways through care for type 2 diabetes for both uncomplicated and complicated cases. Screening for diabetes takes place either during clinical visits with endocrinologists or family doctors, or through outreach campaigns.

Individuals with a positive glucose screen require a confirmatory test (oral glucose tolerance test and/or fasting glucose test) administered by an endocrinologist. Confirmed cases are then eligible for counseling on diabetes management, clinical assessments, and treatment, which can either be provided at PHC clinics or via courses run at residential schools. Patients are either recommended for non-pharmacological treatment (i.e., provided with dietary and exercise advice), or prescribed with oral treatment or insulin. In Poltava region, standard insulin was being provided to diabetes patients for free whereas the costs of oral diabetes medications and some other insulin formulations had to be covered by the patients (a co-payment system for oral drugs was subsequently phased in from 2017 within the Ukraine health reform). Clinical examinations for diabetics are provided at diagnosis and then annually, the main ones being diabetic foot and eye examinations by endocrinologists and ophthalmologists, respectively, and tests for diabetic kidney disease involving nephrologists. Patients are monitored with standard laboratory tests for plasma glucose, glycated hemoglobin (HbA1C test) and cholesterol. Of all diabetics registered in Poltava region in 2016, 47% were reported to have complications (hypoglycemia, diabetic ketoacidosis, retinopathy, cataract, gangrene, and kidney problems).

### Unit costs, coverage, and spending on interventions

We conducted a costing study in one PHC unit with 12 ambulatories and from one secondary level city hospital to collate data on the interventions described in the previous section. Unit costs were calculated as bottom-up costs and included the costs of consumables, salaries, and overheads, with 7% VAT added to drugs and 20% to all other consumables. Salaries were calculated as the average professional rate for 2018 applying to each health cadre, and the prices of consumables were taken from facility invoices. The unit costs associated with the co-payment scheme for drugs were calculated based on the order for border price regulation from the Ukrainian MoH (#148 from 21.01.2019). Costs were converted from Ukrainian hryvnia (UAH) to US dollars (USD) at the mid-2018 exchange rate of 1 USD = 26.3138 UAH [15].

We then gathered estimates of coverage of each of the interventions in 2016 using programmatic reports and expert input gathered during a stakeholder meeting held on 12 February 2019. We estimated 2016 spending by multiplying the intervention unit costs by the number of people covered by each intervention.

### Mathematical model to determine best investments across care cascade

We used the Cascade Analysis Tool or CAT (v1.4.0, ui.cascade.tools), an open-access software application that implements advanced analysis and optimization methods for understanding care cascades, described in detail elsewhere [16]. The CAT is not specific to a particular disease, but rather allows users to specify the pathways through care and how these can be represented as a cascade. Built-in analysis methods can then be used to answer key policy questions, e.g. estimating how available funding should be allocated among available interventions in order to get as many people as possible with successful outcomes. The CAT is based on a compartmental-model-type framework, which implies that the entire population of people estimated to have type 2 diabetes in Poltava region is divided up into one of the states identified in the boxes shown in Fig. 1. At each point in time, it is possible to move between these states according to the transitions shown as arrows on Fig. 1.

We populated the model with data on the state of the type 2 diabetes cascade in Poltava region in 2016 [9], as summarized in Table S1. The flow rates between states are influenced by (a) coverage of interventions that target these flow rates – for example, increasing coverage of screening interventions translates to an increased rate of flow from the “unscreened” to “screened” compartments; and (b) other demographic and epidemiological factors, such as the birth and mortality rates (summarized in Table S1). By using these data in the model, it is possible to attain estimates of the evolution of the cascade under the assumption that the estimated coverage levels do not change, and hence to construct counterfactuals that investigate how the cascade would evolve under different coverage or funding scenarios.

#### Estimating the optimal combination of screening modalities

Screening for type 2 diabetes in Poltava region is conducted both within facilities and via outreach programs. According to available data, 3650 new cases were detected in 2016, with the majority (~ 3350) of these detected through facility-based screening and the remainder (~ 300) via outreach. We investigate scale-up options for screening which would identify 5000 new cases in 2020 under differing assumptions about the positivity rate and maximal attainable coverage of the outreach screening modality. The target of identifying 5000 new cases is chosen to represent a ~ 33% increase over the estimated number of incident cases annually; attaining this target would mean that Poltava region could begin to close the gap between the number of prevalent cases and the number of registered cases.

**Fig. 2:**
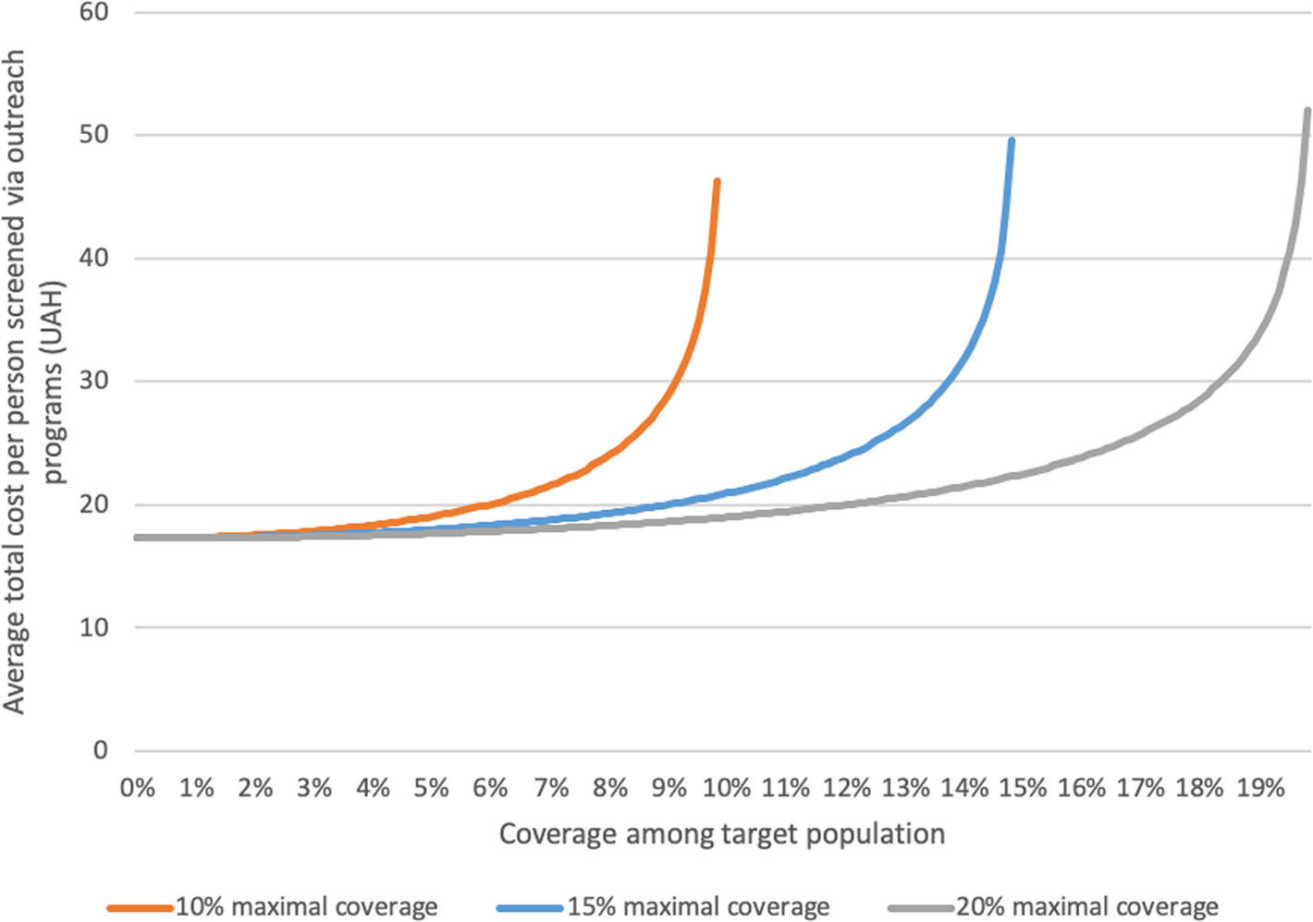
Illustrative average total cost curves for outreach screening programs in Poltava region, Ukraine

To investigate options for scaling up screening, we need to model how the average cost of screening changes as the coverage of screening changes. For this, we use a non-linear cost functions, reflecting the fact that identifying new cases gets more costly as coverage of the outreach programs increase. This type of cost function has been used in numerous other similar mathematical modelling studies [17–19], and is parameterized with two inputs: the unit cost of screening and the maximal attainable coverage, i.e. what proportion of the adult population could feasibly be screened. Since the maximal attainable screening coverage level is unknown, we will treat it as a variable parameter, investigating ranges between 10 and 20% for the outreach screening modality (much of the population would not be deemed at risk, so screening rates are unlikely to increase beyond this). This produces a set of possible nonlinear average total cost functions for the outreach screening intervention, three examples of which are depicted in Fig. 2.

Likewise, the positivity rate of the outreach screening is unknown, so we investigate values between 8 and 16%. These ranges are chosen based on the estimated prevalence of type 2 diabetes in Poltava region, noting that mass screenings are generally not cost-effective [20, 21], so outreach campaigns should be targeted toward likely candidates, in which case the positivity rate is likely to exceed the overall population prevalence. The range here is also intended to account for the unknown number of re-testers, as there is anecdotal evidence to suggest that prevalence in outreach campaigns can be high because of many diabetics use the outreach campaigns as a free of charge glucose measurement. Detection of unique diabetes cases may therefore be lower.

#### Additional investments to improve glucose control

We use the CAT’s inbuilt analysis methods to investigate two interventions that would conceivably improve glucose control outcomes: the oral medication co-payment scheme and enhanced adherence counselling at PHC level or Feldsher posts, the lowest level facility. In each case, we calculate the impact of scaling up the intervention on the costs of meeting patient monitoring needs.

## Results

### Unit costs, coverage, and spending on interventions

In Table 1 we present the estimated unit costs of screening, diagnosis, treatment prescription, and enhanced adherence counseling interventions, along with estimates of the 2016 coverage levels and estimated spend. We also present estimates of the unit costs of providing treatment (insulin and oral) and patient monitoring according to protocols, along with estimates of the number of people in need of each monitoring treatment and monitoring type, and the cost of meeting those needs (disaggregated costs are contained in Table S2).

**Table 1:** Estimated unit costs of screening, diagnosis, treatment prescription, and enhanced adherence counseling interventions and estimates of the 2016 coverage levels and estimated spend; estimates of the unit costs of providing treatment (insulin and oral) and patient monitoring according to protocols, along with estimates of the number of people in need of each monitoring treatment and monitoring type, and the cost of meeting those needs (disaggregated costs are contained in Table S2). Notes: (1) IEC = Information Education Communication; (2) PHC = Primary Health Clinic

	Unit cost (USD)	Estimated number who received service	Estimated spend (USD)
**Screening tests**		45,636	30,003
Facility-based blood glucose test	0.66	41,833	27,503
Outreach/community-based blood glucose test	0.66	3803	2500
**Diagnosis (confirmatory test)**		3344	12,708
Oral glucose tolerance tests	3.80	3344	12,708
**Treatment prescription protocols**		3090	8163
IEC^1^ through residential school/courses	5.76	773	4451
IEC through PHC^2^ clinic staff	1.60	2318	3712
**Adherence counseling**			2085
Enhanced adherence counseling at PHC clinic	0.62	2339	1439
Enhanced adherence counseling at Feldsher post	0.55	1170	646
		**Estimated number in need of service**	**Cost of meeting needs (USD)**
**Treatment maintenance**		40,000	6,097,737
Annual co-payments for oral medication	34.30	31,067	1,065,523
Annual co-payments for insulin	356.56	8600	3,066,413
Annual patient monitoring costs		40,000	1,965,801
*Non-pharma, glucose controlled*	*24.48*	*960*	23,504
*Non-pharma, not achieving glucose target*	*28.28*	*2240*	63,356
*Oral, glucose controlled*	*25.61*	*8460*	216,678
*Oral, not achieving glucose target*	*52.85*	*19,740*	1,043,278
*Insulin, glucose controlled*	*48.97*	*1978*	96,858
*Insulin, not achieving glucose target*	*78.85*	*6622*	522,126

To derive the estimates of the number of people in need of different types of treatment maintenance, we firstly note that around 40,000 people were linked to care in 2016 [9]. Data from the 2018 endocrinology report indicate that around 8600 people were receiving insulin in 2018, and we do not expect that this number has changed significantly since 2016. A review of the diabetes patient files from 2016 indicates that among the insulin patients, ~ 2/3 received insulin only and ~ 1/3 also received oral treatment. The 2016 patient files also indicate that 8% of patients, or 3200 people, received non-pharmacological treatment. Subtracting the insulin patients and the non-pharma patients from the total number of people on treatment leaves 28,200 patients who were receiving oral treatment only, in addition to 2867 who received both insulin and oral treatment. There are therefore an estimated 31,067 oral treatment patients eligible for the oral copayment scheme within the Ukraine health reform.

Patient monitoring costs are generally higher for patients who do not do well on treatment (indicated by not attaining the glycated hemoglobin target level while in diabetes care): 10% higher for patients receiving non-pharmacological treatment, 61% higher for patients prescribed insulin, and twice as high for patients prescribed oral treatment (Table 1).

### Mathematical model to determine best investments across care cascade

After populating the mathematical model with the Poltava type 2 diabetes data, we obtain projections of the estimated evolution of the cascade under the assumption that the 2016 coverage levels indicated in Table 1 are maintained out to 2020 (Figure S1).

#### Estimating the optimal combination of screening modalities

**Fig. 3:**
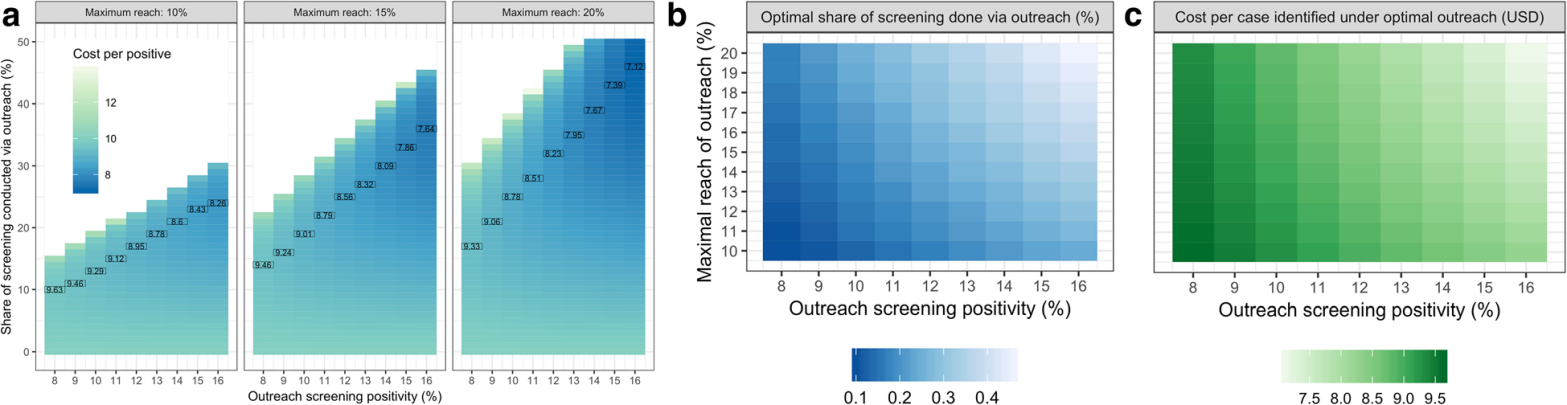
**a** heatmaps indicating the cost of identifying a new case of type 2 diabetes, as a function of the positivity of the outreach screening programs, the maximal attainable coverage of the outreach programs, and the share of all screening activities that are conducted via outreach programs. The black rectangles indicate the minimum cost of identifying a new case for each level of outreach positivity and each maximal attainable coverage level. **b** heatmap indicating the optimal share of screening that should be conducted via outreach as a function of the positivity rate and maximal attainable coverage of outreach screening. **c** heatmap indicating the cost per new case identified as a function of the positivity rate and maximal attainable coverage of outreach screening

The optimal screening scale-up strategy for Poltava region to identify 5000 new cases in 2020 depends on the positivity rate and maximal attainable coverage of outreach screening (where ‘coverage’ here refers to the proportion of adults with type 2 diabetes not already diagnosed who are reached by the program). The more well-targeted and scalable the outreach campaigns are, the greater the share of screening that should be conducted via outreach, as it can reach the large proportion of people who rarely use clinics. Figure 3 displays the results regarding the optimal share of screening that should be conducted via outreach, as well as the cost per positive patient identified, under different assumptions about the positivity rate and maximal attainable coverage of outreach screening. We find that if the outreach program cannot reach more than 10% of the population and only 8% of screening events lead to the identification of a new case, then conducting 10% of screening activities via outreach campaigns (and the remaining 90% via facilities) would be the optimal strategy to minimize the cost of identifying a new case (Fig. 3a). By contrast, if the outreach program could reach 20% of the population and were better targeted (such that 16% of all screening identified a new case), then conducting 46% of screening via outreach be the optimal strategy to minimize the cost of identifying a new case (Fig. 3a). In the former case, the cost per new type 2 diabetes case identified would be USD 9.63 and in the latter it would be USD 7.12 (Fig. 3a). In general, the optimal share of screening that should be conducted via outreach increases as the positivity and maximal reach of the outreach modality increase (Fig. 3b), while cost per positive patient decreases (Fig. 3c).

#### Additional investments to improve glucose control

From Table 1, we note that the annual unit cost of monitoring patients on oral treatment is USD 27.24 lower for patients who have attained sustained glucose control compared to patients who don’t. The cost of annual oral medication co-payments for a single patient is USD 34.30; thus, if providing co-payments improved adherence enough to ensure glucose control, then 80% of the oral medication co-payment scheme could be funded by the state through the reduction in monitoring costs alone.

Using the estimates of annual patient monitoring costs presented in Table 1, we find that a 1 percentage point increase in share of patients achieving sustained glucose control would lead to a reduction in annual patient monitoring costs of USD 10,373 in Poltava region. These additional funds would be sufficient to increase coverage of enhanced adherence counselling by six-fold, enabling almost half of patients to have access to these services.

## Discussion

This study represents one of the first attempts to gather comprehensive, bottom-up costing data for health interventions related to type 2 diabetes case detection, diagnosis, treatment prescription, maintenance, monitoring, and adherence. Analyzing the cascade identified two key breakpoints, the first related to case detection and the second to glucose control while enrolled in diabetes care. The type 2 diabetes patient survey extracting data from cards provided valuable information on patient monitoring and treatment outcomes [9]. Our use of the Cascade Analysis Tool – one of the first applications of this software – allowed us to investigate how to balance facility-based and outreach screening effort in order to improve Poltava region’s efforts at case detection, as well as to investigate how additional funding could be allocated to get more people with sustained glucose control.

Increased diabetes case finding is especially important for groups with low use of health services who miss the screening services offered at clinic level. Data from diabetes screening campaigns in terms of who was actually reached was scarce, although this represents important strategic information to understand screening uptake and yield by population and place. There is considerable scope for targeted promotion of screening during the community mobilization phase of campaigns, targeted screening during campaigns to ensure maximum coverage in older persons and those with increased BMI, and better documentation of participants profile and yield. The literature generally discourages mass screening in diabetes programs as the cost-effectiveness is low [20, 21]. The analyses undertaken here demonstrate the required levels of targeting and reach that outreach screening campaigns would need to have in order to feature as a prominent component of screening scale-up activities and improve entry into diabetes care.

More generally, type 2 diabetes case detection is of crucial importance in numerous settings, and the methods used in this study could usefully be applied elsewhere. A recent review of diabetes cascades in 28 countries found the largest cascade losses at the testing stage, with only 63.4% of those with diabetes having ever been tested [6], which is consistent with other studies that have reported ranges of 24–93% [6, 22–24]. A representative population-level survey in the Ukraine which includes biomarker assessment like blood glucose, blood pressure and body mass would provide crucial evidence on the burden of diabetes, the proportion not taking medicine, and its chief co-factors high BMI and hypertension. To this end, WHO’s STEPwise approach to Surveillance (STEPS) survey [25] has been implemented across Ukraine with results on disparities of the diabetes burden across the regions forthcoming, which will help target control efforts.

Given the extent to which patient costs pose a barrier to effective diabetes treatment [10–12], Ukraine’s move towards improved re-imbursement schemes for oral anti-diabetic drugs is expected to improve glucose control levels. In order to qualify for treatment and re-imbursement schemes, patients are obliged to be examined annually for detecting diabetes-related pathology. Until 2017, metformin and other pre-insulin drugs had to be paid by the patient (averaging at about US$30 per year). The new affordable medicine schemes now offer a set of anti-diabetic drugs free of charge in certain pharmacies. A key improvement to be made is the availability of regular HbA1C testing for diabetes patients to monitor treatment effectiveness: although the test can be conducted via the public sector without requiring an out-of-pocket payment, supplies and access are limited, with the for-profit private sector picking up a portion of the additional demand (at US$4–5 per test, to be conducted every 3 months). The cost of managing diabetes complications is partly covered by patients, with the exception of hemodialysis. Monitoring of the care cascade will reveal whether the reduced patient charges for diabetes medication encourage people to get tested and linked to treatment. The analyses we conducted indicate that around 80% of the costs of implementing the oral medicine co-payment scheme would be covered by the resulting reductions in patient monitoring costs alone.

This analysis had several limitations, both on the data and model side. On the data side: firstly, there was no population-based biomarker survey on the prevalence of diagnosed and undiagnosed diabetes, which would have strengthened this analysis significantly. Secondly, diabetes screening was not systematically reported across PHC and endocrinologists’ services. Thirdly, screening campaign data lacked information about the persons tested and found positive, and whether they were confirmed as diabetes cases. The routine data had the common shortfalls of patient data collected at point of service: incomplete registration of cases, reporting numbers of episodes instead of numbers of persons with episodes, and laboratory data generated in the private sector missing in patient files, while the HbA1C data lacked information on whether they were baseline values at diagnosis or treatment monitoring data, which would bias interpretations on treatment effectiveness, and we could not rule out repeat HbA1C tests being counted as measurements in different patients. Finally, expenditure tracking systems did not work well beyond insulin spend, and unit costs had large uncertainty regarding the time allocated to provide a service. On the model side: firstly, limitations in data availability and reliability can lead to uncertainty surrounding projected results, and these uncertainties were not quantified. Secondly, the model’s estimates could not be validated except by expert review, as there is no historical data that can be used to verify the model calibration. Thirdly, we did not consider the possibility of technical efficiency gains in areas where large volumes of patients are to be served, and which might be expected to lead to economies of scale and reduce unit costs. Finally, the analytical part of the study was conducted during an active phase of the healthcare reform, meaning that some policy aspects were uncertain. Nevertheless, we believe that the analysis conveys a valuable picture of the continuum of care for diabetes cases in Poltava region. As the Ukraine PHC reform progresses, the type 2 diabetes cascade will be re-analyzed to determine how specific interventions have affected care outcomes.

The Cascade Analysis Tool applied in this study is part of a growing toolbox for supporting implementation science, which includes approaches as diverse as return on investment studies [26], economic cost studies [4], machine learning for prediction of disease or complications [27, 28], and resource optimization [29].

## Conclusions

Investments to improve case detection and treatment adherence are the most efficient interventions for improved diabetes control in Poltava region. For a disease like diabetes, which has a growing burden worldwide and consumes a significant portion of health budgets in countries of all income categories, better analytics is increasingly important so programmatic resources can be effectively targeted.

## Supplementary information

**Additional file 1: Table S1.** State of the type 2 diabetes cascade in Poltava in 2016. **Table S2.** Estimated unit costs of providing treatment and patient monitoring according to protocols (US$). **Figure S1.** Model estimates of the evolution of the T2DM cascade in Poltava under the assumption that the spending patterns outlined in Table 1 continue. “Treated” includes both pharmacological and non-pharmacological treatment regimens, and “HbA1C control” includes all of those who were checked for sustained glucose control with HbA1C test and found with HbA1C < 7%.

## Data Availability

All data generated or analysed during this study are included in this published article.

## Abbreviations

NCD: Non-communicable disease
PHC: Primary health care
WHO: World health organization
IDF: International diabetes federation
GDP: Gross domestic product
HbA1C: Glycated haemoglobin
VAT: Value-added tax
MoH: Ministry of health
UAH: Ukrainian hryvnia
USD: US dollars
CAT: Cascade analysis tool
IEC: Information education communication
BMI: Body mass index

## References

[1] WHO. *Diabetes fact sheet*. 2016; Available from: https://www.who.int/news-room/fact-sheets/detail/diabetes.

[2] Twigg, J., *Ukraine’s Health Sector – Sustaining momentum for reform*. 2017: CSIS Global Health Policy Center, August 2017.

[3] NCD Risk Factor Collaboration, Worldwide trends in diabetes since 1980 (2016). A pooled analysis of 751 population-based studies with 4.4 million participants. Lancet.

[4] Seuring T, Archangelidi O, Suhrcke M (2015). The economic costs of type 2 diabetes: a global systematic review. Pharmacoeconomics.

[5] Bloom DE (2011). The global economic burden of noncommunicable diseases (Working Paper Series).

[6] Manne-Goehler J (2019). Health system performance for people with diabetes in 28 low- and middle-income countries: a cross-sectional study of nationally representative surveys. PLoS Med.

[7] Ali MK (2014). A cascade of care for diabetes in the United States: visualizing the gaps. Ann Intern Med.

[8] Stokes A (2017). Prevalence and unmet need for diabetes care across the care continuum in a national sample of south African adults: evidence from the SANHANES-1, 2011-2012. PLoS One.

[9] The World Bank, Type-2 Diabetes Care in Ukraine: Breakpoints and Implications for Action. 2019: The World Bank, Washington, DC. © World Bank. License: CC BY 3.0 IGO.

[10] Матюха ЛФ (2016). Ukrainian experience of health care for patients with diabetes. Wiad Lek.

[11] IDMPS, International Diabetes Management Practices Study wave 7, 2016,*.* Ukraine country report 2017.

[12] Doničová V, Brož J, Sorin I (2011). Health care provision for people with diabetes and postgraduate training of diabetes specialists in eastern European countries. J Diabetes Sci Technol.

[13] Ukrainian MOH, Type-2 diabetes mellitus: clinical protocol for primary and secondary health care level*.* 2012.

[14] School of Public Health of the National University of Kyiv-Mohyla Academy, Ukraine Health Index Surveys 2016 and 2017*.* 2017.

[15] Exchange Rates UK, US Dollar to Ukraine Hryvnia spot Exchange Rates for 2018. 2019.

[16] Kedziora DJ, Abeysuriya R, Kerr CC et al. The Cascade analysis tool: software to analyze and optimize care cascades [version 2; peer review: 3 approved]. Gates Open Res. 2019;3:1488. 10.12688/gatesopenres.13031.2. 10.12688/gatesopenres.13031.2 PMC6944813 31942536

[17] Kelly SL (2018). The global optima HIV allocative efficiency model: targeting resources in efforts to end AIDS. Lancet HIV.

[18] Kerr CC (2015). Optima: a model for HIV epidemic analysis, program prioritization, and resource optimization. JAIDS J Acquir Immune Defic Syndr.

[19] Stuart RM (2018). How should HIV resources be allocated? Lessons learnt from applying optima HIV in 23 countries. J Int AIDS Soc.

[20] Durao S (2015). Evidence insufficient to confirm the value of population screening for diabetes and hypertension in low- and-middle-income settings. S Afr Med J.

[21] Einarson TR (2017). Systematic literature review of the health economic implications of early detection by screening populations at risk for type 2 diabetes. Curr Med Res Opin.

[22] Gakidou E (2011). Management of diabetes and associated cardiovascular risk factors in seven countries: a comparison of data from national health examination surveys. Bull World Health Organ.

[23] Beagley J (2014). Global estimates of undiagnosed diabetes in adults. Diabetes Res Clin Pract.

[24] World Health Organization, Global report on diabetes. 2016.

[25] World Health Organization, The WHO STEPwise approach to chronic disease risk factor surveillance (STEPS) 2019.

[26] Masters R (2017). Return on investment of public health interventions: a systematic review. J Epidemiol Community Health.

[27] Zou Q (2018). Predicting diabetes mellitus with machine learning techniques. Front Genet.

[28] Dagliati A (2017). Machine learning methods to predict diabetes complications. J Diabetes Sci Technol.

[29] Earnshaw SR (2002). Optimal allocation of resources across four interventions for type 2 diabetes. Med Decis Mak.

