# Supplementary material for: Diabetes care cascade in Ukraine: an analysis of breakpoints and opportunities for improved diabetes outcomes
Source: BMC Health Serv Res. 2020 May 11;20:409. doi: 10.1186/s12913-020-05261-y (PMC7212677; doi:10.1186/s12913-020-05261-y)
Supplement: Supplementary file 1 — Additional file 1: Table S1. State of the type 2 diabetes cascade in Poltava in 2016. Table S2. Estimated unit costs of providing treatment and patient monitoring according to protocols (US$). Figure S1. Model estimates of the evolution of the T2DM cascade in Poltava under the assumption that the spending patterns outlined in Table 1 continue. “Treated” includes both pharmacological and non-pharmacological treatment regimens, and “HbA1C control” includes all of those who were checked for sustained glucose control with HbA1C test and found with HbA1C < 7%. [file 12913_2020_5261_MOESM1_ESM.docx]

**Supplementary materials: Diabetes care cascade in Ukraine: An analysis of breakpoints and opportunities for improved diabetes outcomes**

# Table S1. State of the type 2 diabetes cascade in Poltava in 2016

| **Indicator** | **Estimation approach and comments** |
| --- | --- |
| Estimated number with type 2 diabetes | Using the annual regional endocrinology reports 2016, adding the registered type 2 diabetes cases and the estimated undiagnosed type 2 diabetes cases from screening campaigns or expert estimate (adjusted for T2DM using proportion of all diabetes reported as T2DM from annual report). |
| Registered type 2 diabetes patients | Using the total number of type 2 diabetics reported in the annual regional endocrinology reports as registered cases. |
| Number linked to care | Diabetics who are reported as under dispensary supervision, either for non-pharmacological treatment only (lifestyle advice, diet, exercise), or for pharmacological treatment (medication), using annual form #12 data from regional medical statistics units. |
| Number on medication | Using the total number of type 2 diabetics reported in the annual regional endocrinology reports as on medication. |
| HbA1C monitored | Using the reported coverage of HbA1C testing in the annual regional endocrinology reports. |
| HbA1C≤8.0% | Using the data reported on this HbA21C cut-off suggesting reasonable disease control (Diabetes UK, 2017) |
| Glucose controlled (HbA1C≤7.0%) | Defined as persons who have evidence of treatment adherence through reaching normal HbA1C levels of ≤7.0% |
| Chronic morbidity | Using morbidity data in the annual regional endocrinology reports |
| Mortality in diabetes cases or deaths from diabetes-related causes | Using diabetes register data and regional 2015 mortality statistics (the two regions reported diabetes mortality slightly differently) |

# Table S2. Estimated unit costs of providing treatment and patient monitoring according to protocols (US$)

|  | **Non-pharmacological treatment** | | **On oral antidiabetics** | | **On insulins** | |
| --- | --- | --- | --- | --- | --- | --- |
| *Patient achieving treatment target* | *YES* | *NO* | *YES* | *NO* | *YES* | *NO* |
| **Medication** |  |  | **34.30** | **34.30** | **356.56** | **356.56** |
| **Monitoring** | **24.48** | **28.28** | **25.61** | **52.85** | **48.97** | **78.85** |
| *Consultations* | *0.86* | *0.86* | *1.33* | *1.80* | *1.72* | *2.66* |
| *Clinical exams* | *12.44* | *12.44* | *12.44* | *24.88* | *24.88* | *37.31* |
| *Glucose monitoring* | *1.31* | *5.12* | *1.97* | *6.43* | *2.63* | *7.75* |
| *HbA1C monitoring* | *4.18* | *4.18* | *4.18* | *8.36* | *8.36* | *8.36* |
| *Other laboratory tests* | *5.69* | *5.69* | *5.69* | *11.39* | *11.39* | *22.77* |
| **Total** | **24.48** | **28.28** | **59.91** | **87.15** | **405.53** | **435.41** |

Figure S1. Model estimates of the evolution of the T2DM cascade in Poltava under the assumption that the spending patterns outlined in Table 1 continue. “Treated” includes both pharmacological and non-pharmacological treatment regimens, and “HbA1C control” includes all of those who were checked for sustained glucose control with HbA1C test and found with HbA1C<7%.
